# Seawater Accelerated the Aging of Polystyrene and Enhanced Its Toxic Effects on *Caenorhabditis elegans*

**DOI:** 10.3390/ijms242417219

**Published:** 2023-12-07

**Authors:** Tong Zhou, Jiajie Wu, Yun Liu, An Xu

**Affiliations:** 1 Key Laboratory of High Magnetic Field and Ion Beam Physical Biology, Chinese Academy of Sciences, Anhui Province Key Laboratory of Environmental Toxicology and Pollution Control Technology, High Magnetic Field Laboratory, Hefei Institutes of Physical Science, Chinese Academy of Sciences, Hefei 230031, China; 2 School of Graduate Students, University of Science and Technology of China, Hefei 230026, China

**Keywords:** microplastics, seawater, aging, *Caenorhabditis elegans*, toxic effects

## Abstract

Microplastics (MPs) are emerging pollutants and pose a significant threat to marine ecosystems. Although previous studies have documented the mechanisms and toxic effects of aging MPs in various environments, the impact of the marine environment on MPs remains unclear. In the present study, the aging process of polystyrene (PS) in seawater was simulated and the changes in its physicochemical properties were investigated. Our results showed that the surface of the PS eroded in the seawater, which was accompanied by the release of aged MPs with a smaller size. In situ optical photothermal infrared microspectroscopy revealed that the mechanism of PS aging was related to the opening of the carbonyl group and breaking of the bond between carbon and benzene removal. To verify the toxic effects of aged PS, *Caenorhabditis elegans* was exposed to PS. Aged PS resulted in a greater reduction in locomotion, vitality, and reproduction than virgin PS. Mechanistically, aged PS led to oxidative stress, high glutathione s-transferase activity, and high total glutathione in worms. Together, our findings provided novel information regarding the accelerated aging of PS in seawater and the increased toxicity of aged PS, which could improve our understanding of MPs’ ecotoxicity in the marine environment.

## 1. Introduction

Microplastics (MPs) are plastic particles with a particle size of <5 mm [1]. MPs are prevalent in the marine environment, which usually results in “special accumulation zones” in the ocean [2]. Thousands of MPs per square kilometer were detected in the Atlantic and Indian Oceans, whereas >25,000 MPs per square kilometer were found in the Pacific Ocean [3]. The Atlantic and Indian Oceans are less MP-polluted than the Pacific Ocean, but there are thousands of MPs per square kilometer [4,5]. Moreover, MPs have been carried to the North and South Poles by the ocean currents [6,7]. Seawater and glaciers in polar regions contain large quantities of MPs. Considering their environmental persistence and high bioaccumulation potency, MPs are classified as emerging pollutants in marine environments that cause serious environmental damage to marine ecosystems and pose a serious threat to marine life. 

MPs can be divided into primary and secondary types according to their origins [8]. Primary MPs are small particles designed for commercial use in cosmetics and industrial raw materials. As countries worldwide begin to phase out the production and sale of primary MPs, the vast majority of MPs will become secondary MPs [9]. Secondary MPs are formed as a result of the decomposition and aging of plastic products under the action of various environmental factors (such as light, heat, oxidation, hydrolysis, and mechanical action) [10,11]. Plastic aging refers to the changes in the surface physical and chemical properties of plastics after exposure to different environmental conditions [12]. In recent years, the aging mechanisms of MPs have been investigated under various environmental conditions. The irradiation of plastics with UV light caused surface changes in their chemical structures, such as carbon chain breaks, which led to the release of secondary MPs [13,14]. The long-term exposure of plastics to sunlight induced C–Cl bond cleavage in the MPs, thereby forming secondary MPs [15,16]. Thermal aging is another common form of plastic aging. The exposure of aging plastic tea bags, plastic milk bottles, and plastic paper cups to hot water resulted in releasing secondary MPs [17,18,19]. However, the aging of MPs in seawater remains unclear, even though seawater has the highest distributional abundance of MPs.

Over the past decade, the adverse effects of MPs on marine algae have been widely documented [20]. Exposure to MPs slowed the growth and development of microalgal tissues and reduced the chlorophyll content and photosynthetic activity of algae [21,22,23]. High concentrations of polystyrene (PS) affected body size, reproduction, and locomotion in *Daphnia magna* [24]. Wang et al. exposed the larvae of marine shrimp (*Neomysis japonica*) to PS and showed that MPs reduced the feeding efficiency and locomotion of the shrimp [25]. Fish, which are the most abundant animals in the ocean, were also affected by MP toxicity. For example, juveniles exposed to MPs in marine medaka (*Oryzias mlastigma*) could experience delayed development, and MPs accumulated in the ovaries of females, resulting in transgenerational toxic effects [26,27]. Moreover, MPs accumulate in marine organisms through the food chain, ultimately affecting human health [28,29].

*Caenorhabditis elegans* (*C. elegans*) is a suitable model organism for assessing the ecological risks of MPs because of its transparent body, easy culturing, sensitivity to toxins, and compliance with the 3R criteria (reduction, replacement, and refinement) [30]. Toxicity assessments of MPs on *C. elegans* in existing reports have focused on neuro- and reproductive toxicity. Parental PS exposure can trigger a notable reduction in locomotor behavior, eventually leading to hypoactivity in F1–F2-generation worms [31]. MPs are also reproductively toxic, and a variety of MPs, such as low-density polyethylene (LDPE), polylactic acid (PLA), and PS, have been shown to the reduce brood size in *C. elegans* [32,33]. Most current studies on the toxicity of MPs use standard samples (PS beads) for examinations; however, the toxic effects of aged MPs remain unclear [34]. Considering the widespread occurrence of MPs in marine environments, it is imperative to assess the ecotoxic effects of aged MPs in seawater.

Oxidative stress, characterized by an imbalance between the production of reactive oxygen species (ROS) and the body’s antioxidant defenses, poses a threat to cellular integrity [35]. Microplastics, minute plastic particles resulting from degradation or intentional manufacturing, may contribute to oxidative stress through various mechanisms [36,37,38]. The chemical composition of microplastics, including additives and manufacturing residues, has the potential to induce ROS generation upon ingestion [39,40]. Additionally, the physical stress imposed by microplastics on cells, coupled with their role as carriers for adsorbed environmental pollutants, can further contribute to oxidative stress within organisms [41,42]. This complex interplay underscores the importance of investigating the environmental and health implications of microplastic pollution, necessitating comprehensive research to unravel the specific mechanisms and impacts on diverse ecosystems.

In this study, we observed the aging process of MPs in seawater and investigated the changes in the physicochemical properties of aged MPs by using optical photothermal infrared spectroscopy (O-PTIR). We examined the release of secondary MPs using orange fluorescent PS. To assess the toxic effects of virgin and aged MPs, we measured the motility (head thrashes and body bends), viability (group activity counts), and fecundity (germ cell apoptosis and number of oocyte gonads). Considering that oxidative stress is an important mechanism underlying the toxic effects of MPs [38,43,44,45,46,47], we further compared the effects of virgin and aged MPs on nematode oxidative stress-related indicators. This study revealed the aging process of MPs in seawater and elucidated the different toxic effects of virgin and aged MPs. Our findings provided experimental evidence and a basis for the elucidation of the ecological impacts and health risks of microplastics in seawater. 

## 2. Results

### 2.1. Characterization of Virgin and Aged Polystyrene (PS)

**Figure 1:**
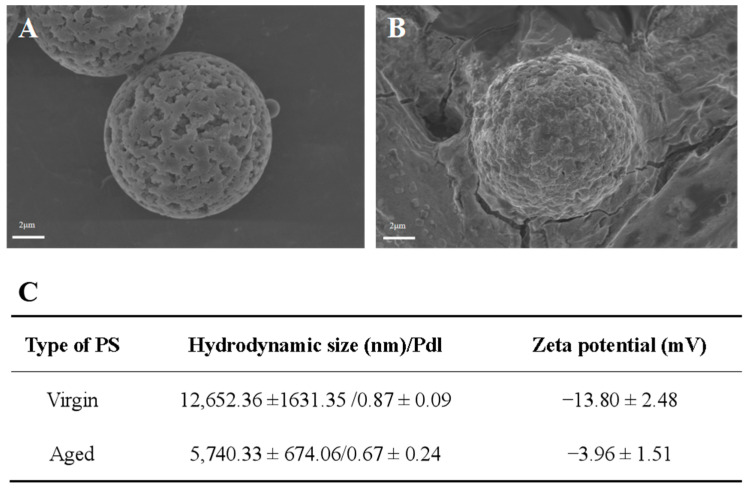
Characterizations of the virgin and aged PS. (**A**,**B**) SEM images of virgin (**A**) and aged (**B**) PS. (**C**) Hydrodynamic sizes and zeta potentials of virgin and aged PS suspensions. All data were presented as means ± SDs of three independent experiments. PdI: polydispersity index.

**Figure 2:**
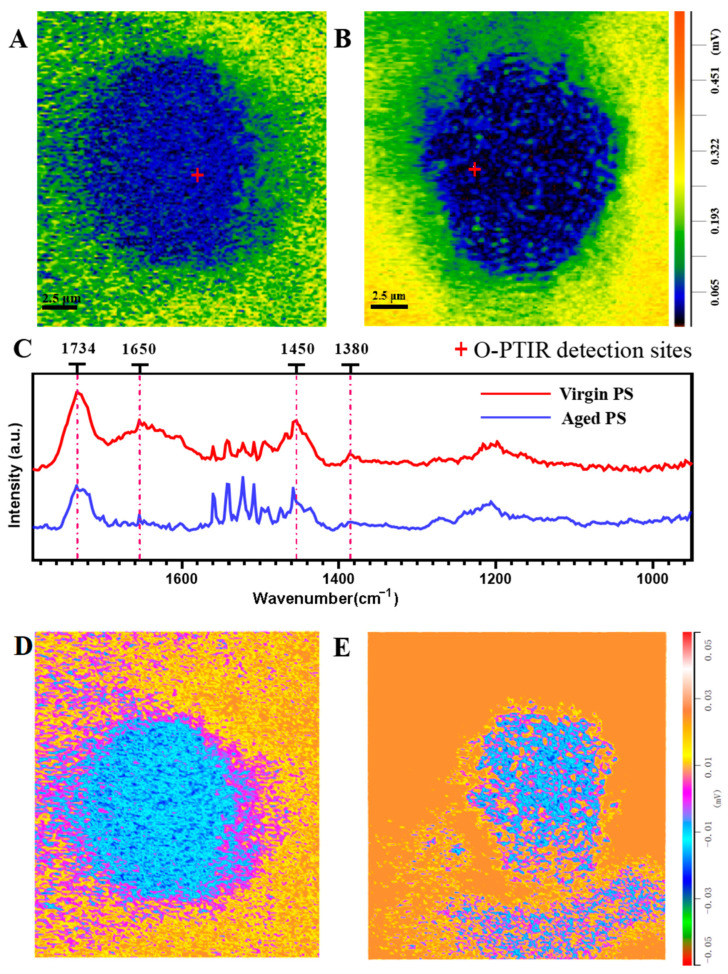
In situ characterizations of the chemical structure of virgin and aged PS by O-PTIR. (**A**,**B**) O-PTIR image of virgin (**A**) and aged (**B**) PS. Virgin PS were spherical while aged PS were irregular in shape. (**C**) Normalized O-PTIR spectra of positions on (**A**,**B**) in situ (a.u., arbitrary unit) were changed in the surface chemical structure of aged PS. (**D**,**E**) O-PTIR tomographic images of virgin (**D**) and aged (**E**) PS considering severe surface corrosion of aged microplastics.

To determine the physicochemical alteration in the surface of PS in seawater, scanning electron microscopy (SEM), dynamic light-scattering (DLS), and optical photothermal infrared (O-PTIR) technologies were used to examine the surface features of virgin and aged PS. Figure 1A,B showed that the virgin PS samples were microspheres with relatively flat surfaces, whereas the aged PS exhibited significant corrosion-altered unevenness on the surface. The DLS technique revealed the different hydrodynamic sizes and dispersion indices (polymer dispersion indices) of the virgin PS (12,652.36 ± 1631.35 nm/0.87 ± 0.09) and aged PS (5740.33 ± 674.06 nm/0.67 ± 0.24) (Figure 1B). In addition, the zeta-potential values of virgin and aged MPs were −13.80 ± 2.48 and −3.96 ± 1.51 mV, respectively. The O-PTIR images revealed that aged PS had an irregular shape, as compared with the spherical virgin PS. Lower infrared signals were detected on aged PS surfaces, possibly accompanied by corrosion (Figure 2A,B). The O-PTIR spectra indicated that the aging process attenuated characteristic absorption peaks in the infrared regions of 1380, 1450, and 1734, while the characteristic absorption peak in the infrared range of 1650 was shifted (Figure 2C). In addition, severe corrosion was observed on the surface of aged PS (Figure 2D,E). These data indicated that PS ages in seawater with surface corrosion and a smaller hydrated particle size, and a lower PDI might result from the release of secondary MPs.

### 2.2. Secondary Microplastics (MPs) Ingested by C. elegans

**Figure 3:**
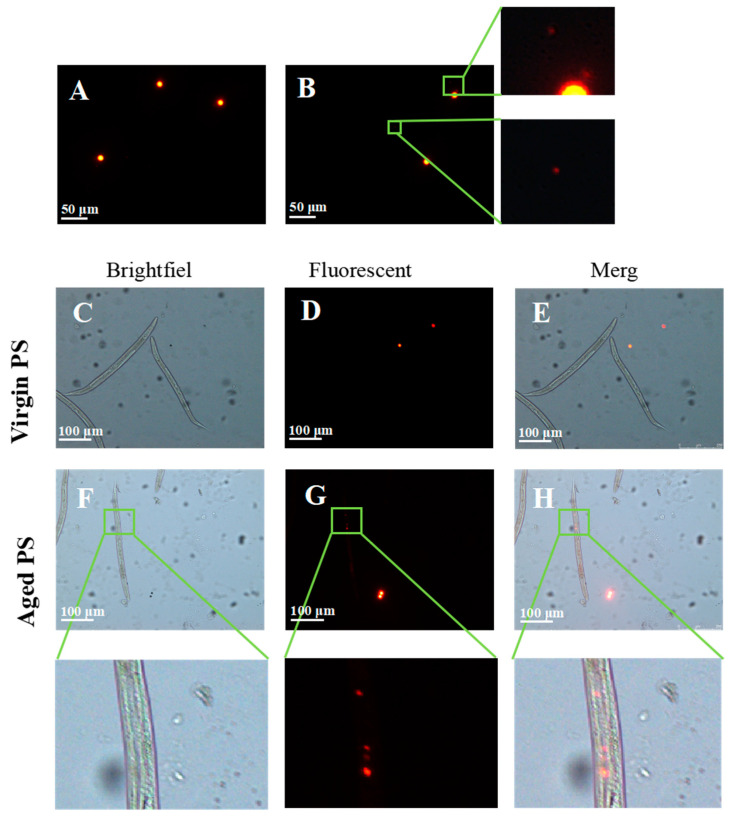
Environmental release and in vivo distribution of aged PS in *C. elegans*. (**A**) Orange fluorescence of virgin PS. (**B**) Orange fluorescence of aged PS. (**C**–**E**) Images of worms exposed to virgin orange fluorescent PS. (**F**–**H**) Images of worms exposed to aged orange fluorescent PS. Aged PS ingested by *C. elegans*.

To confirm whether the aging process of PS in seawater could release secondary MPs, virgin and aged orange fluorescent PS were observed under a fluorescent microscope. While we did not observe any secondary MPs in the virgin PS samples (Figure 3A), secondary MPs with smaller particle sizes were detected in the aged PS samples (Figure 3B). By treating worms with virgin and aged fluorescent PS, we found that virgin PS was unable to enter the worms (Figure 3C–E). In contrast, secondary MPs were observed in the gut of nematodes exposed to aged PS (Figure 3F–H). The results indicated that aged PS could release secondary MPs with irregular shapes and smaller particle sizes in the seawater, and secondary MPs could be ingested by nematodes. 

### 2.3. Effects of Virgin and Aged PS on Locomotion Behavior in C. elegans

**Figure 4:**
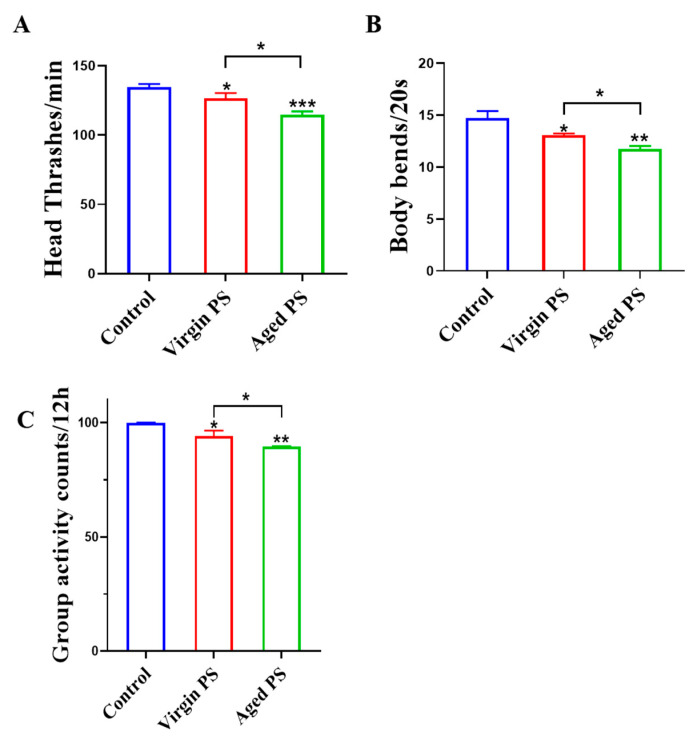
Effects of exposure to virgin and aged PS on locomotion behavior in *C. elegans*. (**A**) Head thrashes. (**B**) Body bends. (**C**) Group activity counts. Error bars indicated ± SEM; * *p* < 0.05, ** *p* < 0.05, and *** *p* < 0.001, compared with the control group.

To compare the neurotoxicity of virgin and aged PS, head thrash, body bending, and vitality were examined in *C. elegans*. As shown in Figure 4A, virgin and aged PS treatments significantly reduced the number of heads thrashed in *C. elegans*. Body bending was also affected by virgin and aged PS, decreasing from 14.70 ± 0.69 in the control group to 13.08 ± 0.14 and 11.73 ± 0.30, respectively. We also applied the worm viability assay system to validate the decrease in motility. The results suggested that virgin and aged PS reduced the motility of nematodes by 5.82% and 10.46%, respectively. These data suggested that aged PS presents greater higher neurotoxicity to worms as compared to virgin PS. 

### 2.4. Effects of Virgin and Aged PS on Germ Cell Apoptosis and Oocytes Development in C. elegans

**Figure 5:**
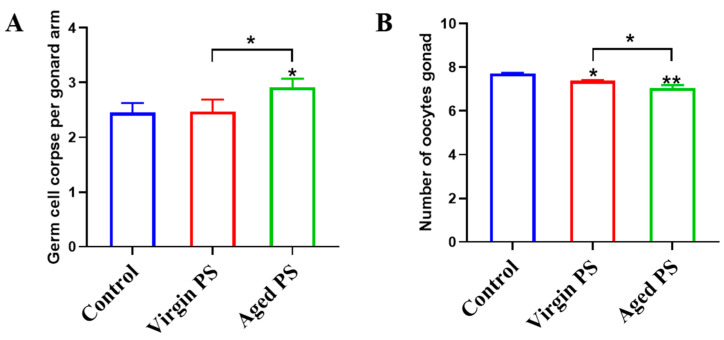
Effects of exposure to virgin and aged PS on germ cell apoptosis and oocytes development in *C. elegans*. (**A**) Average number of germ cell apoptosis. (**B**) Average number of oocytes. Error bars indicated ± SEM; * *p* < 0.05 and ** *p* < 0.05, compared with the control group.

To determine the reproductive toxicity of virgin and aged PS, germ cell apoptosis and oocytes development were examined in *C. elegans*. As shown by Figure 5A, exposure to aged PS significantly increased germ cell apoptosis. As shown in Figure 5B, exposure to virgin and aged PS exhibited a significantly lower number of oocytes in worms compared to the control group. Similar to the results for locomotor behavior, the reproductive toxicity of aged PS was significantly enhanced. 

### 2.5. Aged PS Causes Oxidative Stress in C. elegans

**Figure 6:**
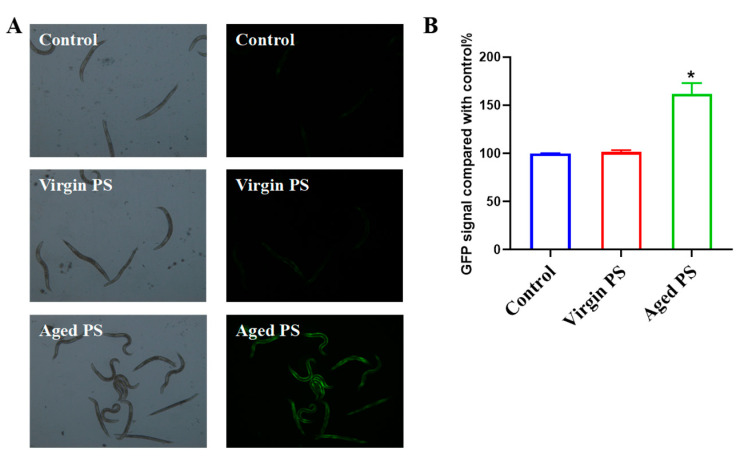
GST expression patterns of CL2166 exposed to virgin and aged PS, respectively. (**A**) Typical brightfield and fluorescence images. (**B**) Intensity of the fluorescent signal was quantitated and presented as mean ± SEM; * *p* < 0.05, compared with the control group.

**Figure 7:**
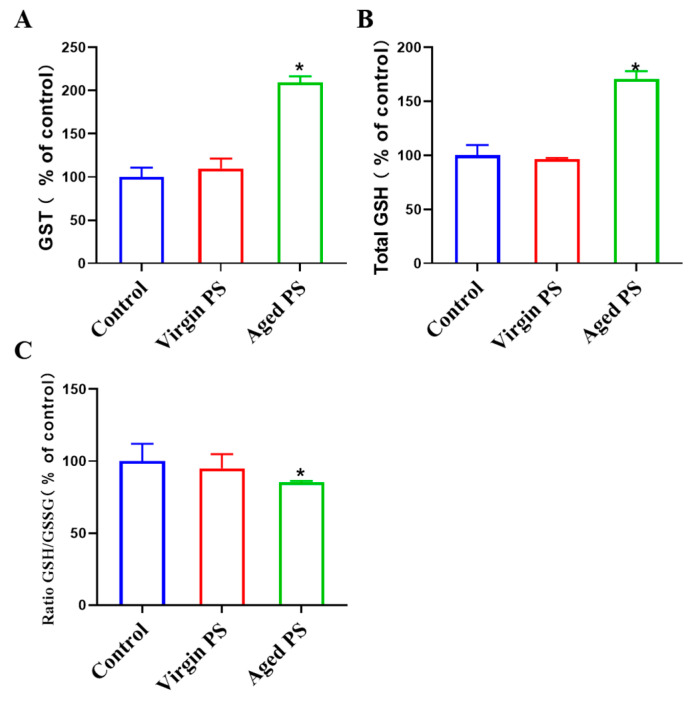
Effects of exposure to virgin and aged PS on GST activity and glutathione level. (**A**) GST activity. (**B**) Total GSH. Aged PS increased total GSH. (**C**) GSH/GSSG ratio. Error bars indicated ± SEM; * *p* < 0.05, compared with the control group.

To explore the mechanisms for different toxic effects between virgin and aged MPs, we examined oxidative stress markers in *C. elegans*. In fact, ROS production was positively related to gst-4 expression according to the previous studies [48,49]. The CL2166 (dvIs19pAF15(gst-4p: GFP::NLS)) strain is often used to detect the occurrence of ROS in *C. elegans*. As shown in Figure 6, a significant increase in fluorescent intensity could be observed in CL2166 exposed to aged PS, as compared to virgin PS. Indicators of oxidative stress, including the activity of GST and the level of glutathione, were examined in Figure 7. Aged PS induced a significant increase in GST-4 (glutathione transferase-4) activity in exposed worms. Meanwhile, the total GSH (glutathione) increased in nematodes exposed to aged PS, though the ratio of GSH/GSSG (oxidized glutathione) decreased significantly due to the increase in GSSG. These results revealed that aged PS could accumulate in the nematode gut and induce oxidative stress. 

## 3. Discussion

The aging of plastics and their release of secondary microplastics have caused great concern in the field of environmental science [50]. Because of their stable chemical structures, the aging process of plastics is very slow under natural conditions [51,52]. However, various environmental factors, such as UV, sunlight, and thermal oxidation, have been found to accelerate the aging process of MPs [53]. In this study, we found that seawater accelerated the aging process of MPs and enhanced their toxic effects on *C. elegans*. 

Previous studies have suggested that the aging process of MPs is usually accompanied by physicochemical modifications on their surfaces, which can change their release pattern [54]. We observed that the surfaces of MPs suspended in seawater were corroded, which was a typical sign of aging. O-PTIR requires no sample preparation and allows the non-invasive simultaneous observation of morphological and chemical structural changes occurring on the plastic surface, making it an ideal method for studying the aging mechanism of MPs in seawater [55]. The O-PTIR analysis showed that carbon bond breaking was the main chemical alteration during PS aging, which was consistent with the other reports [56]. Standard seawater has a pH of 6.2 and there are more hydrogen ions in seawater, which have the potential to accelerate the deterioration of MPs [57]. Mechanical abrasion is involved in the aging process of MPs as well. A large number of salt particles are present in standard seawater, and these salt particles may be adsorbed by MPs and rubbed, which in turn leads to MP aging [58]. 

The toxicity of MPs has been revealed to be related to exposure concentrations and particle size [59]. MPs usually float in the marine environment; therefore, many aquatic organisms accidentally ingest MPs while foraging, but most of these MPs are excreted via intestinal peristalsis. However, with the increase in the exposure concentrations of MPs, the rate of ingestion of MPs by organisms may become higher than the rate of excretion, resulting in the accumulation of MPs in the organisms and causing toxic effects [60,61]. The excretion rate of MPs was inversely proportional to their particle size, with smaller-sized MPs being more difficult to excrete. MPs with smaller particles have shown more significant biological effects on organisms [62]. More importantly, MPs are subjected to continuous aging and degradation because of various factors in real environments. In contrast to the same size of PS plastic microspheres, MPs in natural environments are mostly irregularly shaped fragments or fibers of different sizes. It has been reported that the shape of MPs may also change their toxicity, for irregularly shaped MPs are more likely to interact with the cell membrane and are unfavorable for the exchange of substances between the cell membrane and external nutrients [63]. In a previous study, researchers compared the influence of spherical and irregularly shaped MPs on marine fish and found that irregularly shaped particles could damage the lining of organs more than spherical particles, leading to higher toxicity [64]. Thus, changes in the physical and chemical properties of aged MPs may be responsible for the observed differences in toxicity. 

The toxic effects of microplastics on nematode *C. elegans* mainly concern its reproductive, digestive, and nervous systems. Microplastics induced reproductive toxicity in nematodes at high concentrations, leading to a reduction in egg-laying activity [32,33]. Exposure to polyethylene, polylactic acid, and polystyrene at 100 mg/L, for instance, reduced egg production. Yu et al. found that reproductive toxicity persisted to F4 by exposing nematodes to polystyrene for 72 h over multiple generations [65]. The study suggested that maternal exposure to polystyrene decreased the number of offspring, and even after removing microplastics, these effects could be transferred to subsequent generations, possibly involving epigenetic regulation, such as DNA methylation and histone modification [66]. Microplastic exposure also impacts the growth and development of nematodes, with high concentrations leading to smaller-sized adults [67]. Lifespan is significantly reduced in nematodes exposed to microplastics. Particle size is a determining factor, and the smaller the particle size, the shorter the lifespan [68]. Additionally, microplastics exhibit neurotoxic effects, causing reduced mobility and increased reactive oxygen species (ROS) levels after 24 h of exposure to polystyrene [69]. Similar to reproductive toxicity, neurotoxic effects also present transgenerational impacts. Chen et al. demonstrated this by exposing multiple generations of nematodes (P-F4) to polystyrene, leading to a significant reduction in head swinging and body bending. This indicates that polystyrene-induced neurotoxicity has transgenerational effects, with parental exposure significantly increasing ROS production and lipofuscin accumulation in subsequent generations, highlighting the crucial role of oxidative stress in transgenerational neurotoxicity in *C. elegans* [31].

The toxicity of aged PS on organisms is still largely unknown due to their complicated aging process in the environment. In *Chlorella vulgaris*, aged PS strongly inhibited the growth of microalgae compared to virgin PS [70]. Similarly, aged polyvinyl chloride (PVC) presented more severe developmental toxicity than virgin PVC in Chlamydomonas reinhardtii [63]. Photodegradation increased the toxicity of PS MPs to groupers (*Epinepelus moara*) by disrupting hepatic lipid homeostasis [71]. Chronic exposure to UV-aged PS-PS caused more severe neurotoxicity than virgin PS MPs by affecting neurotransmitter content and related gene expression [72]. To explore the different toxic effects of virgin and aged PS on seawater, multiple endpoints of locomotor activity and reproduction were measured. Our results revealed that the neuro- and reproductive toxicity of aged PS in seawater were considerably enhanced, as compared to virgin PS, which might be closely related to the physicochemical modification of PS aging in seawater. The changes in the physicochemical properties of aged PS might affect its uptake by organisms, resulting in differences in toxicity [73]. MP ingestion by organisms is affected by size, shape, and surface roughness [74,75]. Particle size is considered to be a major factor influencing the uptake of PS by organisms, where smaller PS is more likely to be ingested by organisms [76]. Our results suggested that aged PS could release PS with a smaller size and rougher surface, which might account for the different toxicity levels between virgin and aged PS. 

The decrease in the GSH/GSSG ratio or the increase in the GSSG ratio have been attributed to the oxidative stress induced by various environmental stressors [77]. Oxidative stress occurs as a result of an imbalance between the generation of ROS and the intracellular antioxidant defense system. The cellular antioxidant defense system, including enzymes like glutathione peroxidase (GPx) and glutathione reductase (GR), works to neutralize ROS and prevent cellular damage [78]. Based on our results, it can be hypothesized that the following potential factors may contribute to a decrease in the GSH/GSSG ratio or an increase in the GSSG ratio. GPx functions by facilitating the conversion of GSSG to GSH, utilizing it as a substrate to detoxify hydrogen peroxide and other peroxides, while GR plays a pivotal role in regenerating GSH from GSSG [79]. The accumulation of GSSGs may occur if pollutants or their byproducts directly impede GR activity. Prolonged exposure to pollutants can lead to the depletion of the cellular GSH pool, as GSH is utilized in the process of neutralizing ROS [80,81]. This depletion ultimately results in a diminished GSH/GSSG ratio. In addition, we identified similar phenomena in articles involving exposure to other pollutants (TiO_2_ and polymerases) using the nematode model. The underlying mechanism for this phenomenon may also be associated with the activities of PARP1/PARP2 and SOD enzymes [77,82,83].

The oxidative stress induced by ingested PS *in vivo* is the primary mechanism underlying their toxic effects [84]. The PS particle size was 10 μm in this study, however the 10 μm virgin polystyrene labeled by fluorescence could not be observed in worms under the microscope. This finding was consistent with another previous study, where 6 μm microspheres could not be ingested by the nematodes [59]. The 6 μm polystyrene exerted toxicity effects mainly by affecting nematode locomotion and interacting with bacteria. Aged PS in seawater was found to release irregularly shaped PS with smaller particle sizes, which were ingested and accumulated in the guts of worms to generate oxidative stress. Furthermore, exposure to aged PS increased GST activity, which was related to the self-regulation of worms exposed to external stimuli [85]. When worms were exposed to pollutants, they converted GSH to GSSG by increasing the activity of their enzymes. In this study, we found that seawater accelerated MP aging and its toxic effects were significantly enhanced for locomotion and reproduction, which could be regulate by oxidative stress. This study provided new insights into the aging process and ecological safety of PS in seawater.

## 4. Materials and Methods

### 4.1. Modeling the Aging Process of Microplastics (PS) in Standard Seawater

The PS samples used in this study were 10 μm polystyrene microspheres and 10 μm orange fluorescent PS microspheres, which were purchased from the BaseLine Chromtech Research Centre (Tianjin, China). The standard seawater was a national-level standard substance, which was purchased from Beijing Zhongke QC Biotechnology Co. (Beijing, China). Distilled water and standard seawater in 10 ML amounts were added into conical flasks as the control and seawater aging groups, respectively. The PS samples were suspended in the distilled water and standard seawater for 14 days at room temperature. Virgin and aged PS were obtained by diafiltration using 0.22 μm of AAO membranes and the microplastics were resuspended on the membranes in distilled water. To avoid potential PS contamination, all consumables in contact with the samples were nonplastic. 

### 4.2. Characterization of Physicochemical Properties of Virgin and Aged PS

Scanning electron microscope (SEM): the PS resuspension was ultrasonicated for 1 h to become a well-dispersed suspension, then dispersed in Milli-Q water, placed in a constant-temperature shaking shaker (20 °C, speed 150 r/min, with shaking for 30 min, and vortexed for 3 min to mix it well), then aspirated to 10 L, and dropped onto the copper grid of the electron microscope. Absorbent paper was used to absorb the undried water, and then we placed the copper grid near a table lamp to promote the drying of the water; the PS particle solution was analyzed for the external structure using SEM (ZEISS, EVO MA 15/LS 15, Oberkochen, BW, German). 

Dynamic light scattering (DLS): the PS resuspension was ultrasonicated for 1 h, and after becoming a well-dispersed suspension, it was dispersed in Milli-Q water, and then diluted in Milli-Q, with a final concentration of 1 g/mL placed in a constant-temperature shaker (20 °C, 150 r/min, for 30 min), and then the solution to be obtained was put into the dynamic light-scattering instrument (Malvern, Zetasizer Nano-ZSE, Worcestershire, WR, UK) for detection. Hydrodynamic size: after stabilization for 2 min, the scattering angle was 173°, the temperature was kept at 20 °C, and each sample was detected at least 3 times with an interval of 10 s, and each time, 2 cycles were run without intervals in the middle of each cycle. Zeta potential: after 2 min of stabilization, at a constant temperature of 20 °C, each sample was tested at least 3 times, the interval between each group was set to 5 s, and each test was run for 10 cycles, with no interval between the cycles. 

Optical photothermal infrared: Virgin and aged PS were observed under a mid-IR (1800–800 cm^−1^) mIRage microspectroscope (Photothermal Spectroscopy Corp. Santa Barbara, CA, USA). A tunable, pulsed, four-stage quantum cascade laser device was used as the pump IR source, and a continuous-wave single-frequency 532 nm visible laser provided the probe beam. The quantum cascade laser’s four chips covered a frequency range of 1800–800 cm^−1^. The raw O-PTIR spectra were collected at a spot size of roughly 500 nm and a scanning step size of 2 cm^−1^ and represented the original photothermal amplitude without any manipulations, unless otherwise stated. The O-PTIR spectra were normalized and ratios or overlay maps were produced using PTIR Studio software (v.4.3.7471, Photothermal Spectroscopy Corp. Santa Barbara, CA, USA).

### 4.3. C. elegans Strains and Exposure Concentration

Wild-type (Bristol N2) and CL2166 (dvIs19pAF15(gst-4p: GFP::NLS)) worm strains were provided by the Caenorhabditis Genetics Center (CGC, Minneapolis, MN, USA). All worms were maintained at 20 °C in the dark on worm growth media (NGM) seeded with *E. coli* strain OP50. The worms were synchronized before exposure in all the experiments. The exposure concentrations of PS used in this study were all 100 μg/L based on the results of the pre-experiments not shown here. 

### 4.4. In Vivo Distributions of Virgin and Aged Orange Fluorescent PS in C. elegans

Virgin and aged orange fluorescent microplastics were used to expose L4-stage N2 worms separately for 24 h. A Leica DM4B microscope (Wetzlar, Germany) was selected with a 560 nm excitation peak to track the orange fluorescent microplastics and their released secondary-type microplastics.

### 4.5. Locomotor Behavior Assay

Locomotor behavior was assessed with head forward and body flexion as endpoints in *C. elegans*. After exposure, the worms were washed with M9 buffer and placed on individual non-seeded NGM plates to roam freely for one minute. The frequency of body bend and head thrash movements followed the protocol outlined in previous studies [86,87]. Pharyngeal pumping was scored as the number of contractions and relaxations of the pharynx that occurred in 30 s. Each endpoint was recorded for at least 30 nematodes per group, and 4 replicates were performed. 

### 4.6. Viability Assay

Phylum Tech (Santa Fe, SA, Argentina)’s WMicroTracker™ (WMT) Nematode Viability Assay System was utilized in this study to determine nematode viability levels. L4-stage nematodes were placed in the exposure system in 96-well plates, and the system was used to track nematode viability levels at 30 min intervals up to 12 h. At least 3 independent experiments were conducted with more than 100 nematodes being scored each time.

### 4.7. Fertility Assay

Germline cell apoptosis assay: synchronized L4 worms were treated with PS for 22 h and were then incubated in 25 μg/mL AO for 1 h. The worms were transferred onto OP50 plates for a 45 min recovery period, followed by the scanning of apoptotic cells using a Leica DM4B microscope (Wetzlar, Germany). The apoptotic cells appeared yellow–green after AO staining, representing increased DNA fragmentation, whereas the intact cells were uniformly green. At least three independent experiments were performed and twenty worms were scored for each group. 

Oocytes number assay: the staining procedure was the same as the apoptosis assay. The oocytes of worm gonads were counted using a Leica DM4B microscope (Wetzlar, Germany). At least three independent experiments were performed and twenty worms were scored for each group. 

### 4.8. Activation of the Antioxidative Response

In the transgenic strain CL2166 dvls19(pAF15)gst-4p::gfp::nls, a transcriptional antioxidative reporter enzyme glutathione-S-transferase 4 was co-expressed with a green fluorescent protein (GFP) upon oxidative stress. At the end of PS exposure, CL2166 worms were dropped on empty NGM plates, allowed to dry, and 50–100 nematodes were placed onto slides coated with agar. The slides were examined and GFP fluorescence was captured by the Leica DM4B microscope (Wetzlar, Germany). Two images were captured for each nematode: bright field (to view the animals’ morphologies) and fluorescent. To quantify the intensity of the fluorescence, the two images were stacked using ImageJ (https://imagej.nih.gov/ij/, accessed on 4 December 2023) and, based on each nematode’s morphology, the areas of the pharynx, pharyngeal–intestinal valve, and most anterior intestinal rings were selected for analysis. 

### 4.9. Measurements of Antioxidant Enzyme Activities

GST activity was determined using a Glutathione S-Transferase assay kit (AKPR013M, BOXBIO, Nanjing, Jiangsu province, China). An assay reaction product was placed in a 96-well plate and absorbance at 340 nm was then measured by a fluorescence reader (Spectra Max M2, Molecular Devices, LLC., San Jose, CA, USA). 

Total glutathione and GSH/GSSG were determined using the Total Glutathione (S0052, Beyotime, Shanghai, China) and GSH/GSSG kits (S0053, Beyotime, Shanghai, China). The assay reaction products were placed in 96-well plates, and then the absorbance at 412 nm was measured using a fluorescence reader (Spectra Max M2, USA). The corresponding concentrations were obtained by a comparison with the standard curve performed each time.

### 4.10. Statistical Analysis

A statistical analysis was performed using SPSS Statistic 29 (IBM SPSS Statistics, New York, NY, USA). An exploratory analysis was first conducted to confirm the normal distribution of the data (Shapiro–Wilk test) and variance homogeneity (Levene’s test). The t-test was used to interpret the data for the comparison of two groups, and one-way ANOVA followed by Tukey’s test was used for the multiple group comparison. *p* < 0.05 was used to identify statistically significant differences.

## 5. Conclusions

Microplastics were subject to aging in seawater, as evidenced by surface corrosion and the release of secondary PS. The mechanism of aging was related to changes in their chemical structure, mainly presented in carbon chain breakage. Compared to virgin PS, aged PS could lead to a further increase in neurotoxicity and reproductive toxicity. The reason for the different toxic effects was that aged PS releases secondary PS with smaller and more irregular particle sizes. These secondary PSs could be ingested by nematodes and lead to oxidative stress in nematodes. Our study provided new information on the aging mechanism and toxic effects of PS in the seawater, and a preliminary theoretical basis for understanding the effects of plastic pollution on marine ecosystems and organisms. 

## Data Availability

Data are contained within the article.
